# Utilizing convolutional neural networks for discriminating cancer and stromal cells in three-dimensional cell culture images with nuclei counterstain

**DOI:** 10.1117/1.JBO.29.S2.S22710

**Published:** 2024-08-24

**Authors:** Huu Tuan Nguyen, Nicholas Pietraszek, Sarah E. Shelton, Kwabena Arthur, Roger D. Kamm

**Affiliations:** Massachusetts Institute of Technology (MIT), Department of Mechanical Engineering and Department of Biological Engineering, Cambridge, Massachusetts, United States

**Keywords:** convolutional neural networks, three-dimensional cell culture, confocal microscopy, cell classification

## Abstract

**Significance:**

Accurate cell segmentation and classification in three-dimensional (3D) images are vital for studying live cell behavior and drug responses in 3D tissue culture. Evaluating diverse cell populations in 3D cell culture over time necessitates non-toxic staining methods, as specific fluorescent tags may not be suitable, and immunofluorescence staining can be cytotoxic for prolonged live cell cultures.

**Aim:**

We aim to perform machine learning-based cell classification within a live heterogeneous cell culture population grown in a 3D tissue culture relying only on reflectance, transmittance, and nuclei counterstained images obtained by confocal microscopy.

**Approach:**

In this study, we employed a supervised convolutional neural network (CNN) to classify tumor cells and fibroblasts within 3D-grown spheroids. These cells are first segmented using the marker-controlled watershed image processing method. Training data included nuclei counterstaining, reflectance, and transmitted light images, with stained fibroblast and tumor cells as ground-truth labels.

**Results:**

Our results demonstrate the successful marker-controlled watershed segmentation of 84% of spheroid cells into single cells. We achieved a median accuracy of 67% (95% confidence interval of the median is 65-71%) in identifying cell types. We also recapitulate the original 3D images using the CNN-classified cells to visualize the original 3D-stained image’s cell distribution.

**Conclusion:**

This study introduces a non-invasive toxicity-free approach to 3D cell culture evaluation, combining machine learning with confocal microscopy, opening avenues for advanced cell studies.

## 1 Introduction

Cancer is one of the most significant health challenges in the modern world. According to estimates, in 2020, there were over 19.3 million new cancer cases and almost 10.0 million cancer deaths worldwide.^1^ The goal of personalized medicine is to develop treatment plans based on genetic sequencing of individual patients to identify mutations for which targeted drugs exist. In addition to the genomic classification of targetable mutations, the isolation and culture of *ex-vivo* tissues is an emerging method for drug screening and phenotypic analysis for personalized medicine.^2^ However, real-time monitoring of multiple cell types within thick *ex-vivo* tissues to assess viability and growth in response to treatments is challenging, and immunostaining requires fixation of the tissues.^3^ Critically, changes in the population of immune and tumor cells are a strong indication of immunotherapy efficacy when *ex-vivo* tissues are used as a predictive model for immunotherapy.^4^^,^^5^ This often requires following the progression of excised tumors over time in 3D culture and measuring their response to various therapeutic strategies. For such cases, label-free morphological analysis of cells is advantageous as it offers a rapid, cost-effective method for cell identification compared to labeling cells using dyes. While label-free identification would provide non-invasive, non-toxic identification of cells, most of the work in this field to date has been limited to label-free images in 2D. Therefore, new methods for cell identification in live-cell microscopy are needed to aid in monitoring cells in 3D culture and *ex-vivo* tissue studies and assessing response to therapy.

Machine learning is revolutionizing the image-based diagnosis of disease. Neural networks (NNs) are a class of machine learning models inspired by the structure and functioning of the human brain.^6^^,^^7^ They consist of interconnected processing units, or neurons, organized into layers. Each neuron processes information and communicates with other neurons through weighted connections, which are the parameters for optimization using a training dataset. Previously, researchers used several types of medical imaging training data for machine learning classification to identify cancer. Some example data types include histological images,^8^ non-invasive *in vivo* imaging techniques, such as computed tomography,^9^^,^^10^ magnetic resonance imaging,^11^ positron emission tomography,^12^ single-photon emission computed tomography,^9^ flow cytometry images,^13^ or microscopy images.^14^ In microscopy, machine learning has been employed to perform object detection and classification, enhance image quality and denoising, and recognize cell features, such as membrane and nuclei and image-to-image translation.^15^^,^^16^ Convolutional NNs, or CNNs, are specialized neural networks for image processing. They use convolutional layers to learn spatial hierarchies of features from the input image automatically and adaptively.^17^ Compared to its predecessors, CNNs are particularly effective in classifying images with subtle differences, as they accelerate the training and enhance the final accuracy of the NN program created by taking advantage of patterns in the images they train on.^11^^,^^12^^,^^14^^,^^18^

In bio-imaging, machine learning is used for image classification and feature segmentation.^19^ Label-free cell classification in cell cultures using supervised learning on cell culture in 2D has been reported previously.^14^ However, three-dimensional (3D) images offer a more accurate representation of a cell’s physical morphology within its natural microenvironment. Such 3D images are generally obtained using fluorescent confocal microscopy. Additionally, reflectance confocal microscopy enables imaging of the matrix structure of samples in 3D using the reflectance of light in the far-red spectrum.^20^ Reflectance confocal images have been used as a non-invasive diagnosis method for melanoma,^21^ and CNNs were used for skin texture recognition in reflectance images.^22^ Machine learning-based cell classification in three-dimensional (3D) images of nuclei under brightfield and live counterstaining has been previously documented for tasks like stem cell classification or monitoring embryonic development.^23^^,^^24^ However, there is currently no literature available regarding employing CNN for cell classification in 3D confocal microscopy reflectance images.

Here, we describe a combination of 3D cell imaging using confocal fluorescent, reflectance, and brightfield images, followed by image processing and CNN machine learning as a method to classify cell types in nuclear-stained-only 3D images. The reflected light, in particular, can highlight the intracellular and extracellular matrix structure in 3D without fluorescent tracers. Unlike 2D images, 3D cell images contain information about cell morphologies within the extracellular matrix. We demonstrate that by utilizing three distinct channels, 4',6-diamidino-2-phenylindole (DAPI) (to identify single-cell nuclei), reflectance, and brightfield imaging, we can harness each channel’s unique information to identify cell types successfully. We have developed an image processing workflow designed to segment individual cells based on nuclei positions and reflectance signals within 3D confocal images. We then create, train, and use a CNN for classifying cancer cells and fibroblasts in clusters of cells inside a microfluidic 3D cell culture using DAPI nuclear counterstaining, reflectance, and transmittance (brightfield) signals.

## 2 Materials and Methods

### 2.1 Cell Culture

Normal human lung fibroblasts (NHLFs, Lonza, Basel, Switzerland) were cultured in Fibrolife S2 medium (LifeLine Cell Technology, Maryland, United States) with all provided supplements and used at p6-p9. MDA-MB-231 breast tumor cells, obtained from ATCC (United States), were transfected with RFP-Puro Lentiviral Control Vector (Cell Biolabs, Inc., California, United States). Cells were cultured in DMEM (ThermoFisher, Massachusetts, United States) at 37°C with 5% ${\mathrm{CO}}_{2}$. Tumor spheroids were created by plating NHLFs, stained with CellTracker™ Green CMFDA (Thermofisher, Massachusetts, United States), and RFP-transfected MDA-MB-231 breast tumor cells in a 1:1 ratio at 1M cells/ml in 10 ml of DMEM with 10% FBS on Corning^®^ ultra-low attachment culture dishes (CLS3261 from Corning, New York, United States). These spheroids were collected after 2 days, filtered through a 70  μm strainer to eliminate single cells and spheroids smaller than 70  μm in diameter, and then mixed with fibrin gel solution (2  U/ml thrombin, 3  mg/ml fibrinogen, Millipore Sigma, Missouri, United States) to obtain a concentration of 2000 spheroids/ml. They were then injected into the central gel channel of microfluidic devices (AIM Biotech, United States). Quantifying the number of spheroids was done by aliquoting 50  μl of spheroid suspension solution into a flat-bottom 96-well plate well and counting the number of spheroids inside a 50  μl drop. After fibrin polymerization, the DMEM medium was added to the media channels flanking the gel region. One day after seeding spheroids and gel solution into devices, we changed the media for the device. On day 2, devices were fixed and permeabilized by Triton-X 100 (Millipore Sigma, Missouri, United States), and nuclei were stained by DAPI. As a proof of concept, fixation was done for ease of imaging many devices at the 2-day timepoint, but for live images in future research, Hoechst can be done instead of DAPI.

### 2.2 Image Acquisition

Each microfluidic device has one or multiple sample regions, each accommodating several spheroids. Each tumor spheroid was imaged using a 20× objective (Olympus) on a confocal scanning microscope (FV-1000, Olympus, Japan) with a z-step of 4  μm. For each z position, five types of images (channels) were recorded: blue (excitation wavelength 405 nm)/emission wavelength 461 nm) for nuclei, green (excitation 473 nm/emission 520 nm) for fibroblasts, red (excitation 559 nm, emission 572 nm) for tumor cells, and far red (excitation 635 nm, emission 668 nm, no dichroic mirror) for reflectance light and transmission light from the red laser (559 nm, transmission). We took images with typical width × length dimensions of 463 to 636  μm×382 to 636  μm (pixel size 1.25  pixel/μm), respectively. The height of each z-stack was varied depending on spheroid size, typically between 80 and 100  μm, with a 4  μm step size. Cells near the device’s top or bottom were excluded due to strong confocal reflectance signals at these interfaces.

### 2.3 Single-Cell Segmentation Using Nuclear Counterstaining and Reflectance Imaging

**Fig. 1:**
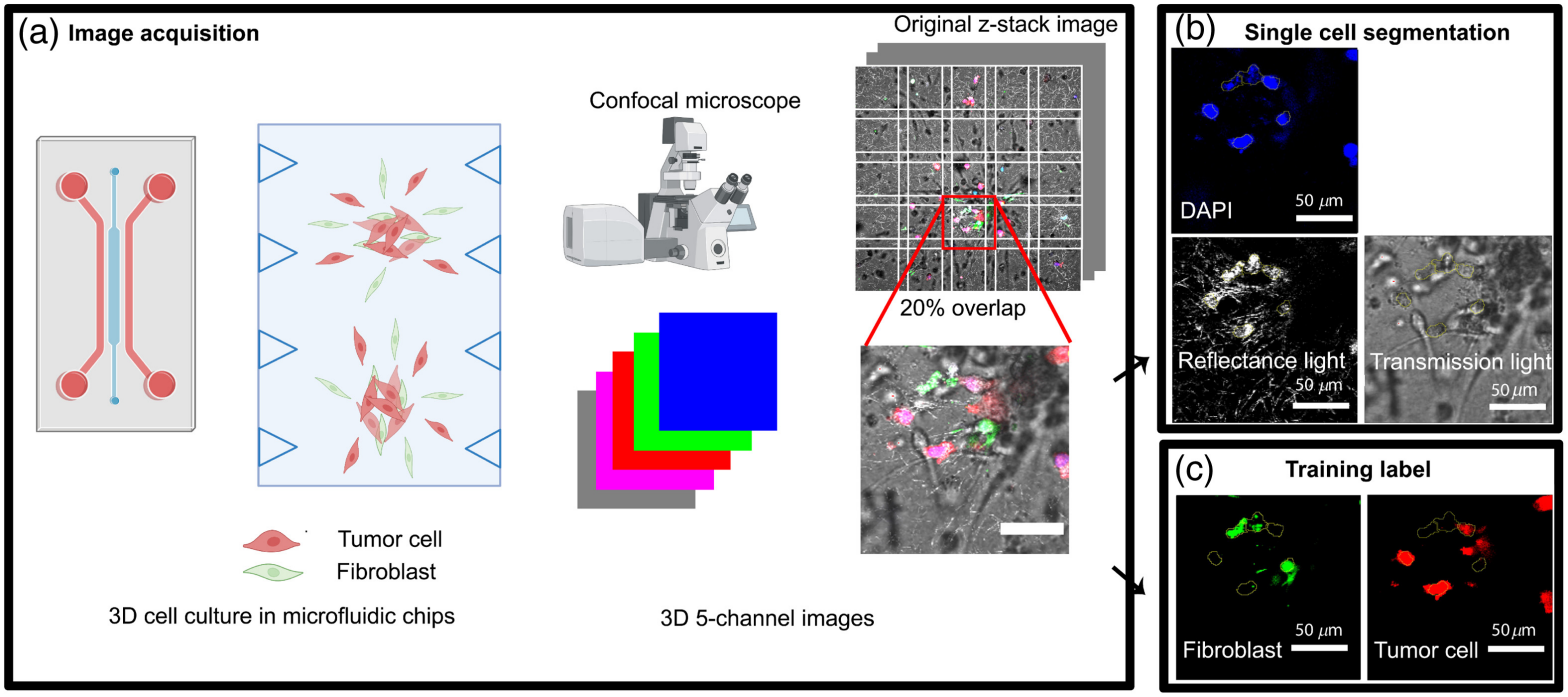
Overview of the image processing and CNNs training and validation procedure. (a) Tumor spheroids are generated by co-culturing tumor cells and fibroblasts within microfluidic devices, followed by 3D cell image acquisition using confocal microscopy. (b) The 3D images are segmented into individual cells using three channels: DAPI, reflection, and transmission signals. These segmented cells are employed for CNN training, validation, and testing. (c) Cell types are distinguished by specific fluorescent signals: green denotes fibroblasts (FB), and red signifies tumor cells (TC).

Next, we developed a FIJI image processing plugin that segmented individual cells in each z-stack multicellular image.^25^ First, each z-stack image was split into smaller tiles (159  μm×159  μm or 200×200  pixels), which had a 20% tile overlap [Fig. 1(a)]. The overlap between tiles allowed the removal of cells that were truncated by the tiling process. Each tile was saved as a multichannel TIFF image, reducing memory usage.

To segment the cells, FIJI’s “marker-controlled watershed” algorithm needed to be used with DAPI and reflectance light (Fig. S1 in the Supplementary Material).^26^ This algorithm treats the input image as a topographic surface, with higher gray values corresponding to greater “altitude,” and simulates a flooding process from seed points. The segmentation procedure comprised the following steps: (1) signal combination: we combined the signal intensities from the DAPI channel (representing the nucleus) and the reflectance channel (highlighting the cell’s cytoskeleton) to form a composite cell representation. Subsequently, Gaussian blur was applied to produce the “blurred cells” image (Fig. S1 in the Supplementary Material, labeled “blurred cells”). (2) Input preparation: based on the blurred image, the original DAPI images, and reflectance images, three input images required for the marker-controlled watershed plugin were generated. “Seed markers” were determined as local maxima from the DAPI image, defining nucleus locations. The “flooding input image,” reflecting cell borders, was derived from a gradient operation applied to the “blurred cells” image. (3) Mask generation: a “Mask” image was generated using the Weka 3D segmentation tool, an integrated machine learning plugin in FIJI. This Mask image identified the location of the cells’ cytoskeleton while distinguishing them from the surrounding fibrin matrix. A classifier was manually trained to differentiate cells from the matrix.^26^ (4) The “marker-controlled watershed” module processed the input images, including “seed markers,” “flooding input image,” and “Mask,” to compute the segmented image, defining individual cells. The reflection image is crucial for defining the area of a cell, as the cell segmentation cannot be done using only the area of the nuclei defined by the DAPI signal (Fig. S1 in the Supplementary Material). Only cells that did not intersect with the image borders were retained after excluding border effects. Additionally, any background signal resulting from potential reflection at the fibrin matrix-glass slide interface, erroneously identified as segmented objects by the “marker-controlled watershed” plugin, was excluded by removing all objects touching the image border, as it consistently covered parts of the image border. The 20% overlap in the tiling process enabled the removal of cells touching the tile’s borders. Subsequently, cells were filtered based on volume, excluding those too small (less than 15 pixels or $37.9\;\mu m^{3}$) or too large (more than 60,000 pixels or $151,686\;\mu m^{3}$). Nuclei with integrated intensities lower than the background level were also excluded. 3D regions of interest (ROIs) encompassing segmented cells were calculated using the 3D manager plugin and recorded for intensity measurement and subsequent 3D reconstruction.^27^ The reflectance, brightfield, and DAPI images of each unlabeled cell were then used as inputs to be classified by the machine learning program [Fig. 1(b)]. Each large image features one or two spheroids. Each tile is a small part of the large image and represents a group of cells within the spheroid or cells that migrate from the spheroid to the nearby matrix. The FIJI macro initially generated over 4852 single-cell 3D images total from the original multicellular images, averaging about 121 single-cell images from each of the 40 multicellular images. Every single cell 3D image had a width and length of 159  μm×159  μm and the height of the original image (80 to 100  μm).

Next, we used DAPI images together with a green or red signal image from each tile to create the ground-truth images of fibroblasts or tumor cells, respectively [Fig. 1(c)]. This process involved the marker-controlled watershed segmentation approach (Fig. S2 in the Supplementary Material). DAPI signal local maxima were still employed to define seed points. For the flooding input image, direct use of the green or red channel image, without additional DAPI input, was preferred due to the channel specificity of the labeled cells compared to the non-specific reflectance image. The mask image [Figs. S2(a) and S2(b) in the Supplementary Material] was obtained by automatically thresholding the fibroblast or tumor cell images using Huang’s algorithm for fibroblasts and Li’s algorithm for tumor cells in FIJI.^28^^,^^29^ Subsequently, segmented fibroblasts and tumor cells were obtained within the reference channels. The cells were obtained through marker-controlled watershed segmentation, using the DAPI local maxima images as markers, the gradient of the red and green images as flooding input images, and the thresholded binary images of the red or green channels as masks. These segmented cells served as ground-truth data for NN training. In some cases, tumor cells and fibroblasts formed dense clusters, making it challenging to define cell boundaries precisely and leading to slight overlaps in ground-truth labeling. We established specific criteria to classify cells as fibroblasts or tumor cells for the training dataset selection. A cell was classified as a fibroblast when its ROI exhibited a fibroblast signal above a defined threshold. Similarly, cells with an ROI with a greater tumor cell signal than the threshold were designated as tumor cells. We introduced an intensity index we designated the F index, calculated as F/(F+C), where F and C represented the binary fibroblast and tumor cell areas within the segmented ROI. An F index exceeding 0.5 denoted a fibroblast, whereas a value equal to or below 0.5 indicated a tumor cell. This criterion ensured that fibroblasts contained more green pixels than red and vice versa.

**Fig. 2:**
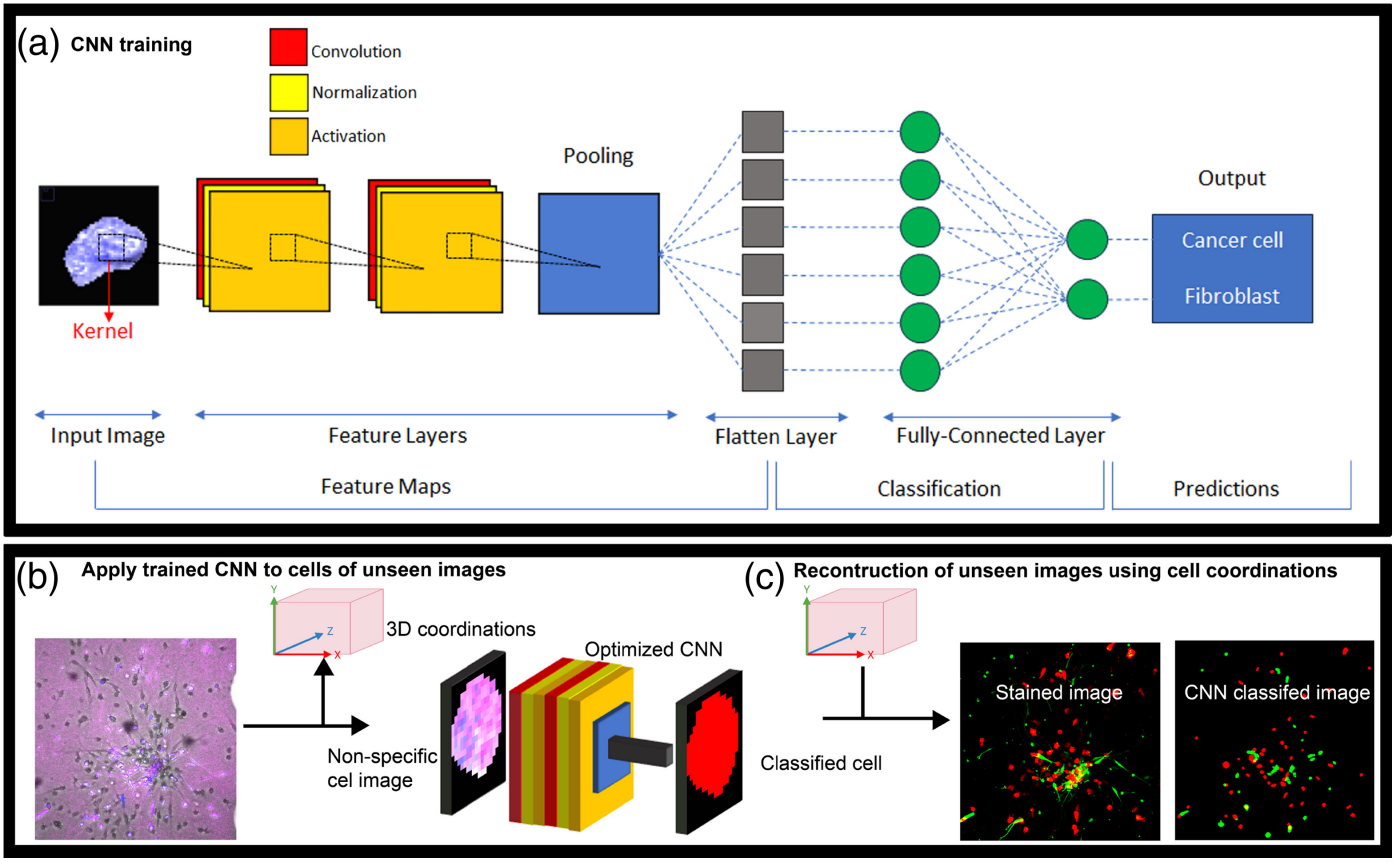
Overview of machine learning training, classification and reconstruction of unseen images (a) CNN architectures and parameters are fine-tuned for optimization, ultimately leading to the selection of the best-performing neural network, K1. (b) The optimized CNN is applied to segment cells in previously unseen images. (c) Image reconstruction process. CNN-classified cells are color-coded according to the cell type and placed at the original XYZ locations of the 3D image to create the recapitulation of the original stained image.

However, there were instances where cells were inaccurately segmented, resulting in erroneous cells with fragments from multiple cells. For example, the comparison of original and segmented cells highlighted in yellow in Fig. 2(a) revealed the complexity of distinguishing fibroblasts from tumor cells, especially in densely packed regions. This complexity arose because each cell in the training dataset needed a clear and exclusive ground-truth label, precluding simultaneous categorization as both a tumor cell and a fibroblast. To address this, we implemented a strategy that assigned segmented cells to ground-truth labels based on both their F index (as previously defined) and their overlap with the most similar ground-truth cell. First, we confirmed a minimum 50% overlap between the cell’s ROI with either the fibroblast or cancer cell staining. Second, we ensured an overlap of over 90% with the most similar ground truth cell.

To evaluate the segmentation accuracy, we compared the number of cells within each 3D image of 159  μm×159  μm×104  μm containing several cells, obtained by cropping from three large images having dimensions of 636  μm×636  μm×104  μm and manually or automatically counting them.

### 2.4 Neural Network Training Dataset Preparation

After segmentation, the 2D slices were converted to matrices through the Numpy library.^30^ As the cells needed to be represented as 3D images, 2D matrices of signal intensity gray value of each z-plane were stacked together as 3D matrices. We standardized individual cell image size to 20×50×50  pixels by adding rows and columns of 0s to the cell images with dimensions smaller than the standard size. Cells larger than these dimensions were removed as they were primarily misshapen, distorted cells, likely errors in the segmentation algorithm. Finally, the 2D matrices were stacked together as 3D matrices representing 3D images of individual cells.

For use in a rigorous test of the model’s generalizing ability, 408 cells from the original 4852 single-cell 3D images were set aside before image augmentation. These cells were from six randomly chosen, original, z-stack images of spheroids not used in training, validation, or prior testing, making them completely separate from the training process. The model’s accuracy was then assessed from its performance on these 408 cells set aside (Table S1 in the Supplementary Material). We next prepared the images for the training process. The remaining 4444 3D single-cell images were first curated by removing all images where the F index and overlap method disagreed, as those cells had an ambiguous ground truth. Only 277 out of 4444 segmented cells (constituting 6.2% discordance and 93.8% accordance) had uncertain ground-truth labels, often due to weak fluorescence in either the red or green channel. Consequently, we successfully assigned ground-truth labels to 4167 single cells. Next, to ensure the data is not biased toward either cell type, we removed the tumor cells with the lowest confidence until there were equal numbers of fibroblasts and tumor cells. Therefore, we randomly selected 1144 tumor cells from a pool of 3023 and included all 1144 fibroblasts. The 2288 images were copied three times and rotated 90 deg, 180 deg, and 270 deg as a form of image augmentation to increase the amount of data available to use fourfold. The resulting 9152 3D images were then arbitrarily divided into training, validation, and testing cells. The training cells are the cells that the model “learns” from, optimizing the weights it uses to classify images into cell types. The validation cells are used as a metric to determine how well the model can generalize on the cells it has not seen already during training. The strict testing cells evaluate the model’s final performance on cells it did not see during training. Each 3D matrix representing a 3D cell image from each of the three data image types (Reflection, transmission, DAPI) was stacked together into a 4D matrix to feed into the machine learning program.

### 2.5 Creating the CNN Models

We developed our models and trained and evaluated their performances using the Keras and TensorFlow libraries.^7^ We hypothesized that the order of the randomized image sets presented to Keras and TensorFlow could have some effect on the machine learning’s performance. Therefore, we designed a bootstrapping program that automated the training under the same parameters, each with a different set of images for their training, validation, and testing datasets (Table S1 in the Supplementary Material). For each training run, we put each image in the training, validation, and testing datasets into a different random order before starting the multiple training epochs for that dataset. We performed 25 training runs, selecting the model with the best validation loss.

The CNN model was built on a modified version of the VGG-16 architecture.^18^ The CNN model was a traditional 3D CNN with a batch normalization layer, an ReLU activation function layer, an Adam optimizer, and a Max Pooling 3D layer [Fig. 1(d)]. The training run for this machine learning model created our best-performing NN, which we dubbed K1. We used the “Adam” optimizer, with an initial learning rate of 0.0001, an exponential scheduled learning rate decay, a batch size of 16, a kernel size of 5, a pooling size of (2,2,2), and 16 CNN filters.

The loss function was set to the categorical cross-entropy cost function

Loss=∑i=1nyi*log(y^i).

To evaluate the performance of our classification method, we compared the model’s prediction to the ground truth cell type for each segmented cell. Then, we recapitulate a 3D image of the original multicellular image using machine-learning classification and the 3D manager plugin by assigning the classification from the NN to each ROI and placing them in the 3D coordination within the original image to recapitulate the 3D original image.

## 3 Results

### 3.1 Image Processing of Reflectance and Counterstaining Signals to Achieve Single-Cell Segmentation in Confocal 3D Images for Convolutional Neural Network Training, Validation, and Testing

The experimental workflow involved co-culturing tumor cells and fibroblasts in a low-adhesion well plate, facilitating the formation of heterogeneous spheroids. These spheroids were subsequently transferred into microfluidic chips and cultured within fibrin gel for an additional day. During this time, they began to disperse before being fixed and imaged by confocal microscopy [Fig. 1(a)]. The confocal microscopy images comprised three channels: blue DAPI for nuclear staining, reflectance, and transmission light for non-specific extracellular and intracellular matrix structures, and brightfield images capturing cell morphology. Additionally, two fluorescence channels, green and red, were utilized to distinguish fibroblast and tumor cell labels.

In order to streamline subsequent image processing tasks and reduce computational load, the original images were divided into smaller tiles with a 20% overlap. Single-cell segmentation was performed using a marker-control segmentation algorithm in ImageJ, primarily employing the DAPI and reflectance signals [as illustrated in Fig. 1(b)]. Following the identification of ROIs corresponding to individual cells, the combination of DAPI, reflectance, and transmission signals within each ROI constituted the non-specific cell image used for training the CNN. The label assigned to each cell was determined based on the relative intensity of the green and red signals.

Out of the entire dataset of 4852 cells, a subset of 408 cells (comprising 8.4% of the total dataset), within 8 multicellular images were reserved for strict testing purposes. The remaining cells were curated and subjected to rotational augmentations to construct the training, validation, and testing datasets, facilitating the optimization of the CNN model. Detailed cell counts for each group are provided in Table S1 in the Supplementary Material. Following the optimization process, our most proficient neural network model, denoted as K1, was established [as shown in Fig. 2(a)]. Detailed CNN optimization results are described in Sec. 2.2; see below.

Subsequently, K1 was employed to classify unseen cell images from the strict testing set, as illustrated in Fig. 2(b). To generate stained images based on the CNN-classified cells, we combined cell coordinates and the classification results from the CNN. This reconstruction process resulted in the recapitulation of the original stained image [Fig. 2(c)].

**Fig. 3:**
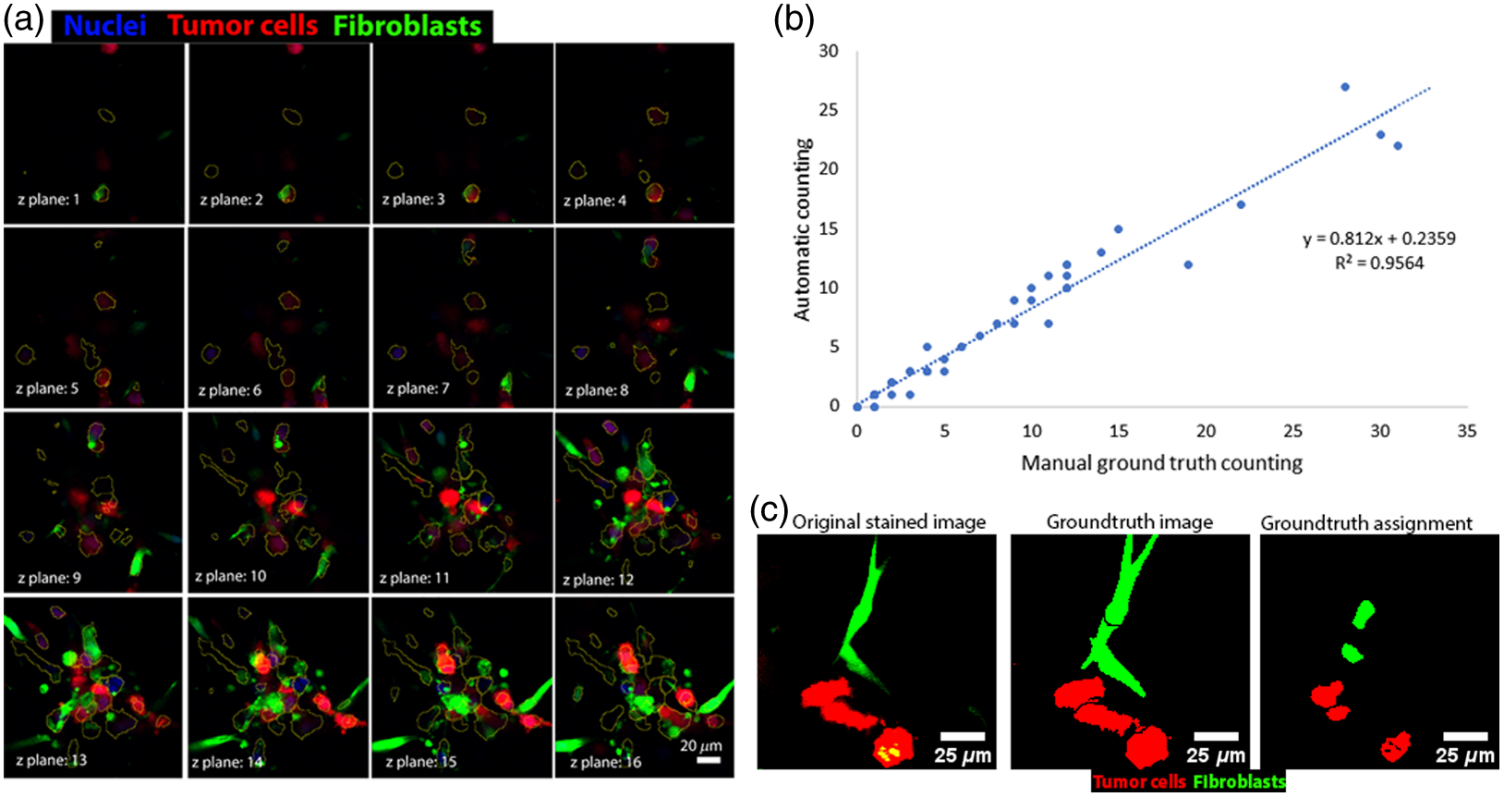
Single-cell segmentation and ground-truth assignment. (a) Visual comparison of 3D automatic segmentation of single cells to reference cells in a cluster of cells. Cells that touch the border of the images are not considered. (b) Comparison of automatic counting with blind manual counting across various sampled 3D images. The manual counting was conducted prior to the automatic counting in a one-side blinded setup. (c) Comparison of the original staining, the ground-truth segmentation image, and the cell type assignment of the segmented cells, excluding cells that touch the border, based on the F index (see Sec. 2). Segmented cells are slightly smaller than the original stained cells.

To assess the segmentation performance, we compare the segmented cells (in yellow) to the green and red reference channels of the same image, which display the positions of fibroblasts and tumor cells [Fig. 3(a)]. To quantify the performance of the segmentation FIJI plugin, we counted the total number of cells manually in 12 randomly selected tile images of 159  μm×159  μm×104  μm and compared them with the number of cells segmented in a blinded manner. We plot the correlation graphs between the manually counted number of cells versus the one obtained with the automatic segmentation protocol [Fig. 3(b)].

As a result, we obtain a strong correlation between the number of cells obtained by automatic and manual counting in each individual tile image ($R^{2}=0.95$). We found that the ratio between the number of automatically counted and manually counted cells was 0.84. We verified the segmentation accuracy by comparing the segmented cells and the labeled images and observed that the automatic segmentation method did not pick up any false-positive cases. The program lost some cells, however, especially when they were tightly aggregated due to quasi-overlapped DAPI signals of different nuclei, causing heteroskedasticity. After segmentation, we assign the ground-truth label to each segmented cell based on the intensity of the green and red channels of the image [Fig. 3(c)]. Besides the marker-controlled watershed operation, several other 3D Segmentation algorithms in Fiji, such as 3D spot segmentation and 3D watershed were tested but visually, the segmentation accuracy of these methods are less than marker-controlled watershed because the latter uses the information from the reflectance images, which contain both signals from matrix and cells that are needed for the detection of cell borders.

### 3.2 Optimized NNs Achieve 67% Classification Accuracy in the Testing Dataset

The average training time per epoch was 330 s, and the median epoch of minimum validation loss was 9 (Table 1).

**Table 1:** Optimized NN results. The modified VGG-16 model was trained in several training runs, and the best model was selected from these runs.

**Epochs**	30
**Testing accuracy**	70%
**Strict testing accuracy**	67%

We tested our best NN (K1) against a set of 3D cell images from our 1632 cells rotated from 408 cells from the strict validation data set, which was omitted from the NN training. The NN achieves a maximum accuracy of 70% and a median accuracy of 67% (Table 1).

### 3.3 Reflectance Image is Essential for the Training of CNNs

To identify what data were most important to the machine learning’s predictions, we trained the machine learning on datasets where the training data were restricted and found the optimized machine learning’s corresponding classification accuracy. We compared the machine learning’s performance when the training dataset used has either all three channels (DAPI, reflectance, and transmission signal), a combination of two channels, or only a single channel. We also restricted the number of cells the machine learning was allowed to train on.

**Fig. 4:**
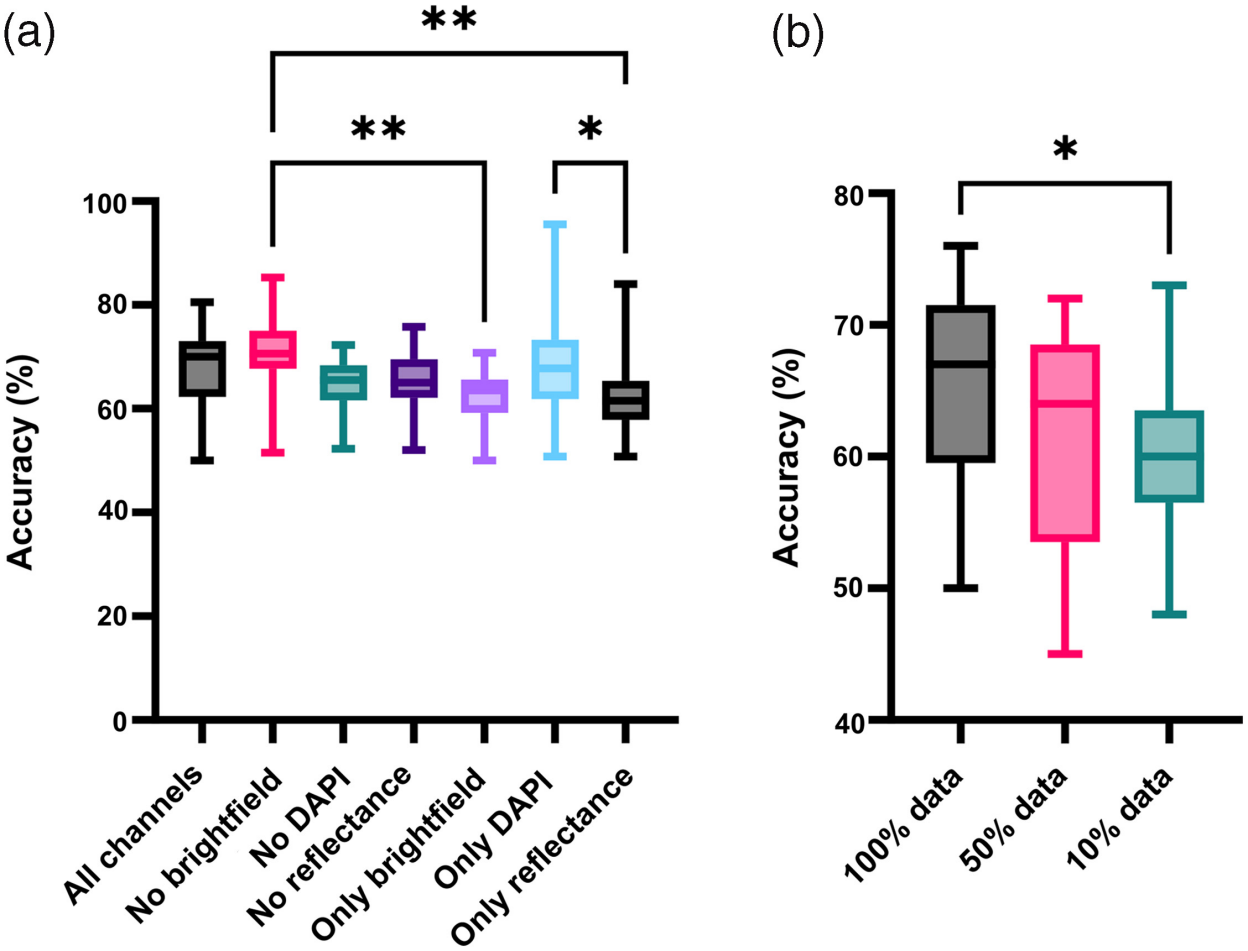
Input features and data set tests. (a) Comparison of validation accuracy between different combinations of channels DAPI, reflectance, and brightfield as model inputs on the test set. From left to right: all three image types DAPI, reflectance (R) and brightfield (BF), DAPI and R, BF and DAPI, R and BF, only BF, only DAPI, and only R. (n=25 to 27). (b) Impact of the training set size on classification accuracy (n=27, 26, and 16, respectively, one-way ANOVA with Tukey with multiple comparisons test; *, P<0.05, **, P<0.01).

When we average the classification accuracy of all optimized NNs obtained with each training dataset, we can confirm that the accuracy of the NNs trained with three channel-dataset is among the highest, together with DAPI and reflectance combination (∼70% median), or at least equal to the one obtained with either one or two channel(s) [Fig. 4(a)]. Although the DAPI-reflectance dataset led to slightly higher accuracy (70%) compared to the three-channel dataset (67%), we still use the three-channel dataset for validation and testing because the brightfield image of cells contains biological information that we might be able to extract in the future.

To test whether increased data would significantly improve our machine learning’s accuracy, we removed the image augmentation of rotation and cut the number of cells used in training by 90% and 50%. As expected, the accuracy was much lower with smaller data sizes, stemming from insufficient examples causing overfitting and reduced generalizability. The loss of accuracy when the data is reduced by 50% is small, implying additional data will have a minor improvement in accuracy [Fig. 4(b)].

### 3.4 Recapitulation of 3D Image Succeeded in Reproducing the Original Cell Position and Type

**Fig. 5:**
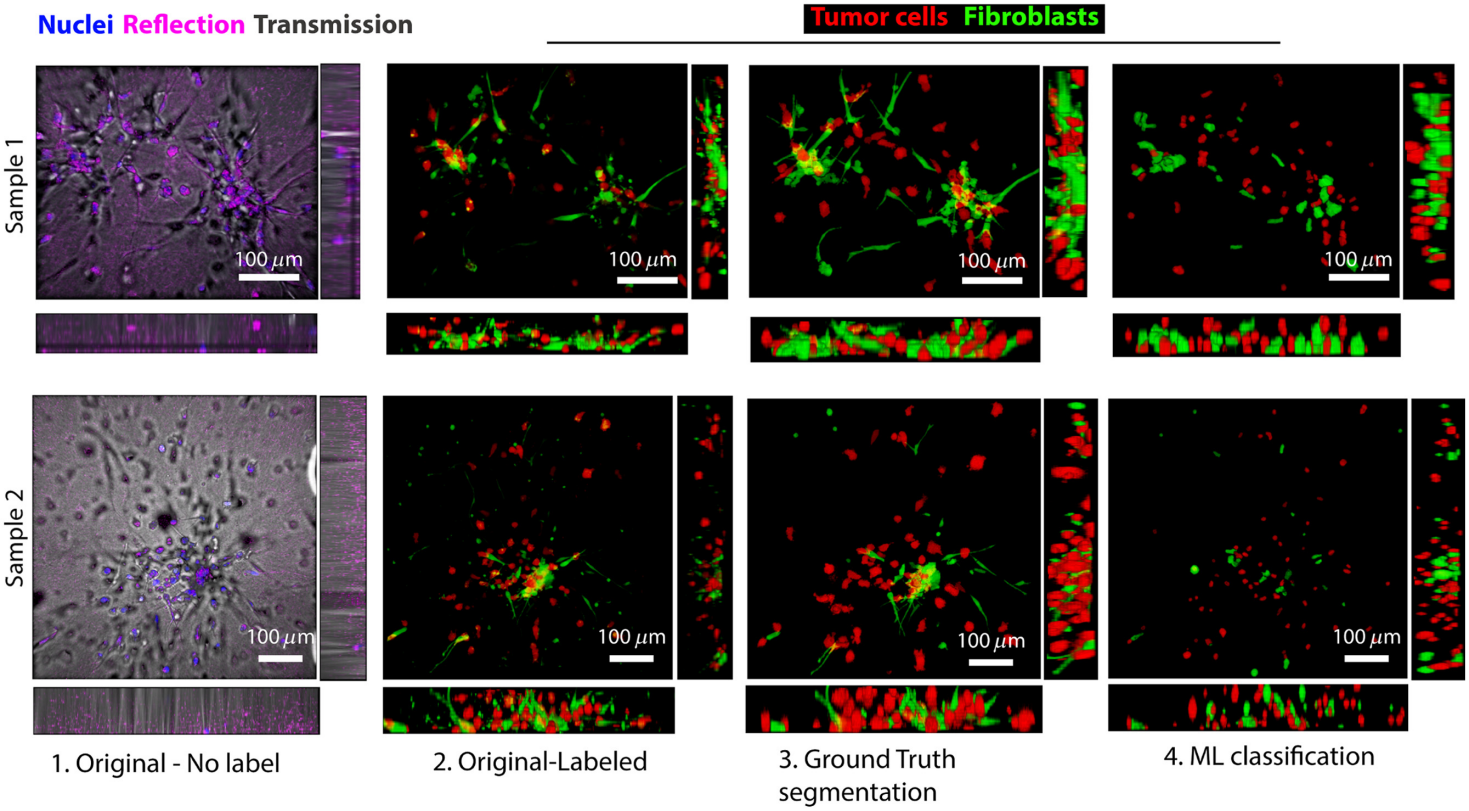
Recapitulation of the initial 3D images using machine learning cell classification. Each row represents one representative sample. From left to right: 1. Original non-labeled images are the projection of three-channel z-stack images: nuclei stained by DAPI, reflectance, and transmission images of the confocal microscope. 2. The original labeled image of cells. 3. Ground-truth image obtained by identifying cell type (either fibroblast or tumor cell) based on the intensity of the green and red channels of the original-labeled image. ROIs without a nucleus are excluded. 4. The recapitulated 3D images by machine learning.

By keeping track of which cells we used from our rigorous test dataset, we recapitulated what the predictions would have looked like as an image for each 3D ROI. We then compared the nuclei, reflectance, and transmission original images, the fibroblast and tumor cell channels of the original images, and the corresponding segmented ground-truth image with these images (Fig. 5). The recapitulated 3D images demonstrate that our method combining cell segmentation and machine learning can reflect the cell distribution of the stained 3D tissue culture images.

## 4 Discussion

This study combines 3D cell culture, 3D cell segmentation, and machine learning techniques to create a new automated approach for classifying 3D confocal cell images using only reflectance, transmittance, and nuclear-counterstained images. Post-processing based on automated FIJI macros and Python code processed these images, providing suitable single-cell inputs to a machine-learning model. Our work demonstrates the power of combining techniques from bioengineering and machine learning, in particular, the creation of multiple types of 3D images of cells (i.e., reflectance, brightfield, and DAPI) to create a NN with the ability to classify cells of 3D images that are indistinguishable by eye with 67% accuracy. This modest accuracy primarily results from regions containing densely packed cells (Fig. 4), where segmentation challenges arise due to the non-specific nature of reflectance imaging. Several avenues exist for enhancing this accuracy, including improving image quality by increasing magnification and resolution, enhancing image processing techniques, expanding the training dataset, and optimizing the network architecture. Additionally, the application of transfer learning methods offers promising potential for further improvements (see below). We also developed an image processing protocol that recapitulates the original tissue by mapping AI-classified cells back to their relative position within the tissue.

As a direct application, using this method, we can segment and classify cells within live 3D images of tissue culture samples that have Hoechst staining instead of DAPI. The accurate labeling of cells to create the ground-truth for training and evaluating the NNs during training should be a particular focus here. Future research should perform the classification of cells within a patient’s tissues.

We believe future models capable of classifying multiple cancer cell types will require similar optimization and may also benefit from exploring some of the leading-edge machine learning techniques, such as transfer learning.^31^

In our proof-of-concept study, we opted for the marker-controlled watershed algorithm to perform the segmentation of individual cells within 3D images.^26^ This choice was based on the algorithm’s classical approach, which provides precise control over the segmentation process for cells, encompassing both nuclei and cytoplasm. Notably, this method relies on nuclei images as seeds for cell segmentation, ensuring that each cell is associated with one and only one nucleus. However, in future applications, we intend to explore machine learning-based segmentation techniques like U-Net and Stardist.^32^^,^^33^ These advanced methods have the potential to enhance segmentation accuracy.

Transfer learning starts with a NN pre-trained on the appropriate subject matter (e.g., cell images), and then training this NN in the specifics of the image library for the classification exercise. Our research could not apply this technique due to the lack of a generally available initial NN trained on 4D matrices (three channels of 3D images). Future research should explore explicitly creating this type of initial NN and exploring its effect on improving classification accuracy. Applying models pre-trained on 3D cell culture classification to the analysis of patient-derived tissues through transfer learning could further validate and extend the applicability of our approach.^34^ Moreover, to further enhance our classification accuracy in future studies, we plan to compare our results with those obtained from various CNN architectures, such as AlexNet, Inception, and Resnet.^35^^,^^36^ This comparative analysis will provide valuable insights into the performance and suitability of different CNN models for our specific cell classification tasks.

## 5 Conclusion

This study has successfully demonstrated the potential of machine learning for cell classification in nuclei-counterstained-only 3D cell culture images. Utilizing a microfluidic device, we cultured heterogeneous populations of tumor and non-tumor cells in 3D, applied 3D cell segmentation, and employed deep learning to categorize label-free single-cell images as either cancer cells or fibroblasts, achieving a classification accuracy of 67%. The information derived from neural network-based classification allows us to reconstruct aspects of cellular spatial distribution. This reconstruction aids in estimating the migration behaviors, morphological characteristics, and interactions among cell populations over extended culture periods.

This methodology, when extended to encompass various cell types, holds promise for diverse applications. Standardized multicellular 3D images can serve as input for an automated process capable of accurately and cost-effectively classifying unlabeled live cells. This approach can be employed for imaging live *ex-vivo* tissues or organoids in 3D cell culture, enabling the classification of different cell types within the tissues through our image processing and machine learning protocol. Consequently, we can monitor interactions among various cells within the tumor microenvironment and their responses to therapeutic interventions in a non-invasive manner. This study represents a pivotal proof-of-concept, potentially paving the way for long-term investigations into real-time cellular events within 3D cell culture systems for drug discovery and personalized medicine applications.

## Supplementary Material



## Data Availability

Original multicellular images are available at: https://fairdomhub.org/studies/1247. FIJI Image processing code is available at: https://github.com/huutuannguyen/3DCellCulture.git. CNN and preprocessing Python code is available at: https://github.com/npietraszek/MIT_Cancer_Identification_Research_Project.
