# Supplementary material for: Utilizing convolutional neural networks for discriminating cancer and stromal cells in three-dimensional cell culture images with nuclei counterstain
Source: J Biomed Opt. 2024 Aug 24;29(Suppl 2):S22710. doi: 10.1117/1.JBO.29.S2.S22710 (PMC11344342; doi:10.1117/1.JBO.29.S2.S22710)
Supplement: Supplementary file 1 [file JBO_029_S22710_SD001.pdf]

Utilizing convolutional neural networks for discriminating cancer and stromal cells in three-dimensional cell culture images with nuclei counterstain

Huu Tuan Nguyen¶, Nicholas Pietraszek¶, Sarah E. Shelton, Kwabena Arthur, Roger D. Kamm*

Massachusetts Institute of Technology, Department of Mechanical Engineering and Department of Biological Engineering, Cambridge, MA, 02139, USA

¶ These authors contributed equally to this work.
* Corresponding author. E-mail: rdkamm@mit.edu

Supplementary figures

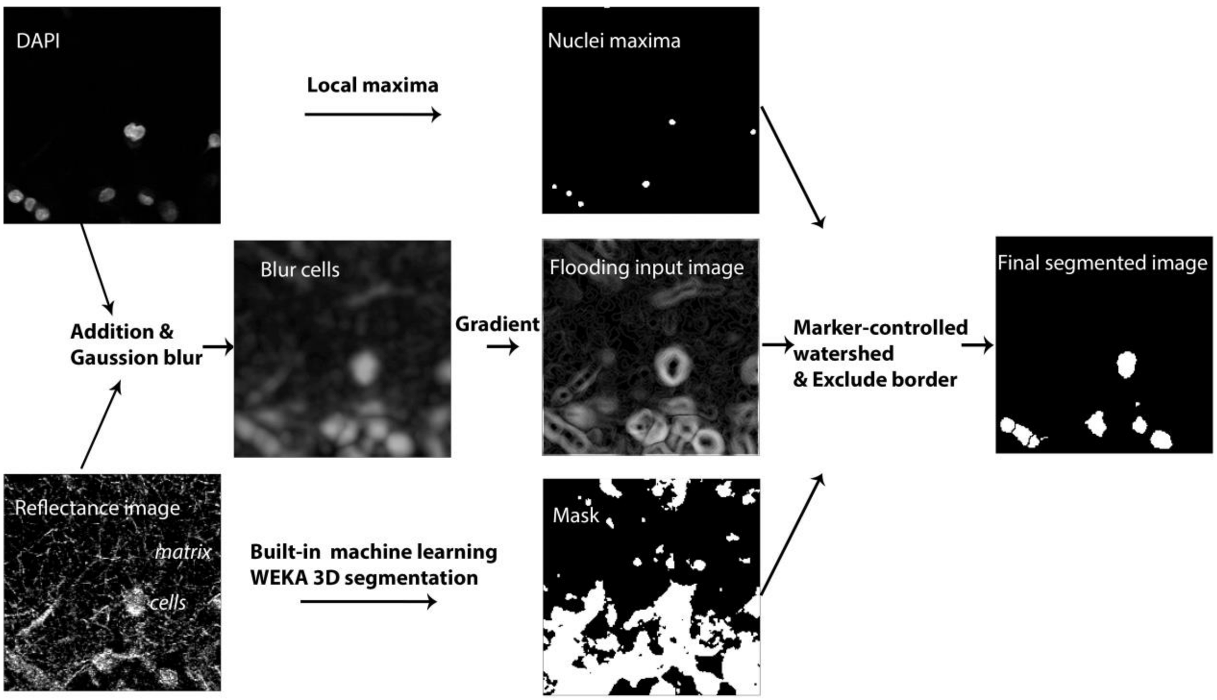

Fig. S1 Segmentation method using DAPI and reflectance image. From images of the DAPI channel, we detect local maxima to obtain the position of nuclei in the image "Seed markers," i.e., nuclei maxima. Also, we perform the addition of DAPI and reflectance images, then apply Gaussian blur operation to obtain image "Blur cells." Next, gradient operation was applied to the "Blur cells" image to obtain the border of cells. The marker-control watershed considers the input image, i.e., the "Flooding input image" in our case, as a topographic surface with higher gray values representing higher altitude and simulates its flooding from the seed points, i.e., the "Seed markers" in our case. We also apply a built-in machine learning WEKA 3D segmentation on the reflection image to identify cellular structures from the extracellular matrix. Finally, we perform the marker-controlled watershed operation with the input images "Seed markers," "Flooding input image," and "Mask" and exclude the particles at the border to obtain the final segmented image.

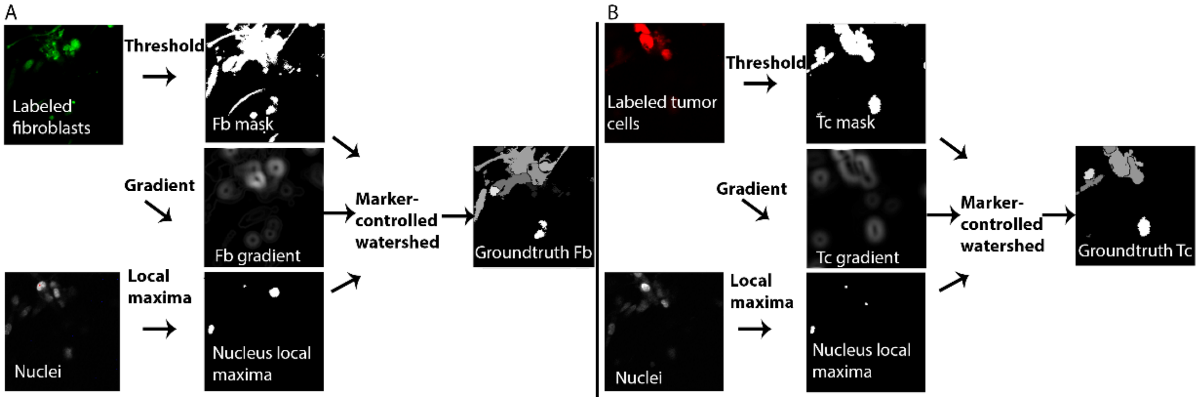

Fig S2 Image processing for training data. Single fibroblasts (A) and cancer cells (B) were analyzed using the marker-controlled watershed algorithm. The steps shown in each sequence are, from left to right: the fluorescent signal for fibroblasts/tumor cells and the DAPI image of cell nuclei; corresponding images obtained using the FIJI's 'thresholding,' 'gradient,' or 'local maximum' operations; and the 'marker-controlled watershed' operation that produced segmented ground-truth fibroblasts and tumor cells. Nuclei maxima are identified at one focal plane of a z-stack only.

Supplementary table

Table S1 Data selection for training: Original, unrotated cells are curated for creating a training dataset through various steps: undetermined ground-truth cell removal, strict testing cell removal, balancing the number of tumor cells and fibroblasts, and rotation for data augmentation. After rotation, cells are allocated to training, validation, testing, and strict testing data.

Total Unrotated Cells	Undetermined Ground-truth-Cell Removal	Strict Testing Cell Removal	After Balancing	After Rotation	Training Data	Validation Data	Testing Data
4852	4444 (4852-408)	4167 (4444-277)	2288 (4167-1879)	9152 (2288 × 4)	8352	400	400
